# Environment and CVD: moving from Risk Prediction to Risk Management

**DOI:** 10.1007/s11883-025-01375-y

**Published:** 2026-01-13

**Authors:** Tasveer Khawaja, El Hussain Shamsa, Sanjay Rajagopalan

**Affiliations:** University Hospitals, Harrington Heart and Vascular Institute, Case Western Reserve University School of Medicine, Cleveland, OH USA

**Keywords:** Cardiovascular prevention, Residual cardiovascular risk, Air pollution, Exposome

## Abstract

**Purpose of review:**

We attempt to provide a framework for cardiovascular risk assessment related to environmental pollutants to enhance awareness of risk posed by environmental risk factors and highlight approaches for risk intervention.

**Recent findings:**

Indisputable links between environmental exposures and cardiovascular outcomes exist. Although many of these relationships are well studied, such as air pollution, evidence continues to emerge regarding others, including noise, light, the built environment, and temperature.

**Summary:**

When the totality of the impact from environmental exposures are considered under the paradigm of the exposome, their health impact and disease burden form a considerable part of mitigatable residual cardiovascular risk. This risk can be attenuated by policy and, to a lesser extent, individual level actions. By harnessing artificial intelligence, we can move to integrated risk exposure analysis and target those at the highest risk with interventions.

## Introduction

The Lancet Commission on Pollution and Health has deemed environmental pollution a leading cause of chronic diseases and premature deaths globally [1, 2]. The widely cited Global Burden of Disease (GBD) estimates that around 9.0 million total deaths are directly attributable to environmental pollution, accounting for 16% of all annual deaths, a number similar to deaths caused by smoking [3, 4]. The World Health Organization reported a global excess mortality of 12.6 million in 2012 as being due to unhealthy environments [5, 6]. More than 50% of the risk of most environmental risk factors are likely due to the detrimental cardiovascular and metabolic effects that they confer [7–11]. Environmental exposures are seldom experienced in isolation and are often substantially influenced by factors such as the built and natural environment and interact with a variety of extrinsic factors and co-pollutants, other hidden chemical exposures, noise, and light [7, 12, 13]. Many contemporary studies are now focused on the concept of the “exposome,” or the sum totality of exposures and their health impacts.

When the totality of impact from environmental exposures is considered, their health impact and disease burden could exceed worst scenario estimates. From a population health and policy perspective, identifying vulnerable populations—defined by age, sex, race, socioeconomic factors, and susceptible individuals who may be more sensitive to the effects of environmental risk factors—is imperative. There is also a substantial need for awareness of how environmental factors affect health, the incorporation of these factors into risk assessment models, and, importantly, the creation of pathways to communicate this risk to individuals and populations. Finally, a move towards developing an evidence base for preventive therapies and measures that mitigate environmental risk is a compelling need of the hour.

In this review, we attempt to provide a framework for this vision of moving from mere risk assessment, to enhanced awareness of risk posed by environmental risk factors, including relevant communication approaches and finally, approaches for risk intervention.

## Environmental Risk Factors

**Fig. 1:**
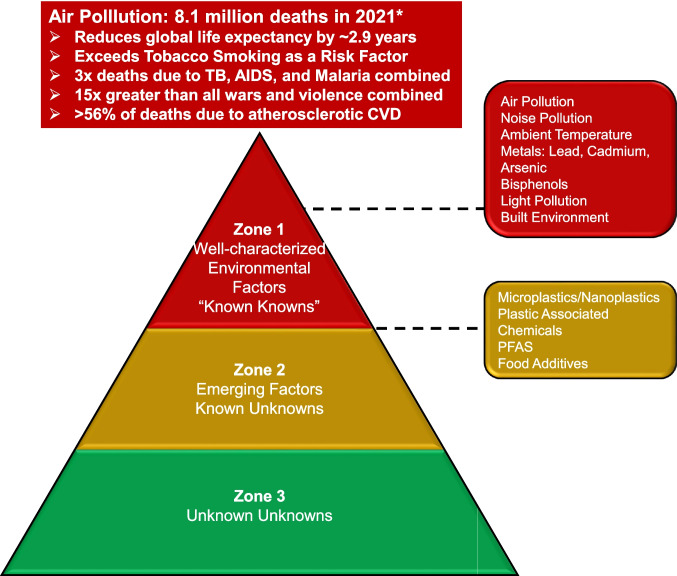
The zones of evidence linking pollution and cardiovascular disease. This figure was adapted from Landrigan et al. Lancet 2018;391(10119):462-512

The known health effects of many pollutants are likely the tip of the iceberg as we understand the exposure risk of only a small number of pollutants [14]. Most exposures remain unknown with unknown risk, while the small set of emerging risk factors lack delineation of dose response relationships and other aspects of toxicology (Fig. 1). Importantly, while chemicals in the air, water, and soil may potentiate risk factors for cardiometabolic disease, non-chemical environmental factors such as temperature, noise exposure, mental/psychosocial stress, socioeconomic factors, electromagnetic fields, occupational risks, built environments, agricultural methods, and man-made climate and ecosystem change may co-segregate with air pollution. Taken together, these risk factors may work synergistically to amplify their association with cardiovascular (CV) events [15, 16]. An emerging body of evidence links many chemical pollutants with cardiovascular and metabolic disorders like hypertension, ischemic heart disease, and diabetes [17, 18]. Environmental pollution, lifestyle factors (e.g., smoking, nutrition, physical activity), climatic conditions (e.g., high temperature and humidity, UV radiation), social exposures (e.g., social isolation, economic distress, work strain) are all considered parts of the exposome that may significantly impact inflammatory cascades, redox homeostasis, and the circadian rhythm [8, 12, 19–23].

## Air Pollution

There is a large body of epidemiologic evidence indicating that air pollution plays an important role in global cardiovascular mortality and morbidity [2, 24]. Air pollution is the most significant chemical environmental health risk, with particulate matter with a diameter ≤ 2.5 µm (PM_2.5_) and ozone responsible for 8.8 million global premature annual deaths [2, 25]. These estimates are expected to increase due to improved exposure–response functions and more accurate high-exposure level studies from highly polluted megacities [26]. Globally, poor air quality accounts for more than 6 million deaths per year, although recent estimates put this number well above 9 million deaths with a global mean overall loss of life of 2.9 years. In comparison, tobacco smoking results in a 2.2 year attrition, highlighting the importance of ambient air pollution as a global risk factor [27]. Importantly, an estimated 5.13 million excess deaths per year globally are attributable to ambient air pollution from fossil fuel use and therefore could potentially be avoided by phasing out fossil fuels [28]. In 2019, the total economic cost of air pollution exceeded $8 trillion US, surpassing 6.1 percent of the global annual GDP [29]. However, these costs do not fully reflect the socioeconomic burden of air pollution, including productivity loss due to sick days and benefits for those with consequent disability.

Cardiovascular diseases account for the majority (~ 60%) of the excess in morbidity and mortality due to PM_2.5_ and likely most environmental risk factors [2, 30]. A host of large epidemiologic studies conducted across the world have established a consistent association between air pollution exposure (especially PM_2.5_) and increased cardiovascular risk factors (e.g., hypertension, diabetes), surrogate markers of disease (e.g., coronary artery calcium), and cardiovascular morbidity and mortality (e.g., ischemic heart disease, stroke, and heart failure) [7, 8, 10, 11, 13, 31–35]. Most of these studies have been conducted in adults, as summarized extensively in previous meta-analyses, reviews, and consensus documents [10, 36–38]. Although air pollution poses smaller relative risks than traditional risk factors (e.g., tobacco, hyperlipidemia), it presents an enormous public health threat due to its ubiquitous nature, involuntarily impacting billions of people daily. Low- and middle-income countries suffer by far the greatest consequence [39]. Health organizations across the world, including the American Heart Association (AHA) and European Society of Cardiology (ESC), have therefore deemed PM_2.5_ as an important causal risk factor for cardiovascular morbidity and mortality [40–42]. Both short (hours-to-days) and long-term (months-to-years) exposures increase cardiovascular risk. However, the former does so by only 1% whereas the latter does so by roughly 10% (per 10 µg/m^3^ of PM_2.5_). The health risks posed by chronic exposures are thus an order of magnitude greater than those induced by acute episodes. This core concept has pivotal ramifications for public health, especially for children and young adults potentially facing a lifetime of higher exposures [14].

## Noise

The impact of noise on cardiovascular health has been reviewed previously [43]. Chronic exposure to environmental noise, particularly from traffic, aircraft, and industry has been increasingly recognized as an independent risk factor for cardiovascular disease. Noise activates the sympathetic nervous system and the hypothalamic–pituitary–adrenal axis, leading to elevated stress hormones, endothelial dysfunction, oxidative stress, and systemic inflammation [34, 44]. These pathways contribute to hypertension, arrhythmias, ischemic heart disease, heart failure, and stroke [7]. The risk is dose-dependent, with night-time noise and long-term cumulative exposure posing the greatest harm, especially in individuals with co-existing risk factors (such as obesity, diabetes, or air pollution exposure) where synergistic effects amplify cardiovascular risk [43].

## Light pollution

Light pollution—particularly exposure to artificial light at night (ALAN)—disrupts circadian rhythms, impairing cardiovascular and metabolic health [45–47]. Nighttime light exposure disturbs sleep, and chrono-biological entrainment, triggering autonomic activation, systemic inflammation, insulin resistance, and endothelial dysfunction. Epidemiological studies have linked ALAN to elevated risks of coronary heart disease, including myocardial infarction, stroke, obesity, hypertension, and diabetes [48].

## Built Environment

The built environment impacts cardiovascular and metabolic outcomes across multiple scales and has been extensively reviewed [9, 49, 50]. At the macroscale, metropolitan planning determines access to housing, employment, healthcare, recreation, and essential services, shaping multiple socioeconomic gradients of risk. The mesoscale of transportation and mobility networks—including highways, transit, and pedestrian or bicycle infrastructure—affects exposure to pollution and opportunities for physical activity. At the microscale, neighborhood-level design features such as street layout, safety, greenery, and public space influence stress, social cohesion, and walkability [9, 49]. Collectively, these factors can either promote cardiovascular resilience or reinforce vulnerability to disease.

## Temperature

Exposure to both heat and cold increases cardiovascular morbidity and mortality in a non-linear U- or J-shaped pattern, with risk rising as temperatures deviate from an optimal threshold (minimum mortality temperature) that varies by geography and acclimatization [51]. While cold has historically caused more total cardiovascular deaths due to its higher frequency, climate change is shifting the balance toward heat-related mortality, which carries greater per-day risk. High ambient temperatures are associated with a range of acute cardiovascular outcomes, including stroke, heart failure, arrhythmias, and cardiac arrest [52]. A global systematic review and meta-analysis found that each 1 °C rise in high temperature was associated with a 2.1% increase in cardiovascular mortality and a 0.5% increase in cardiovascular morbidity [53]. Risks are particularly elevated for stroke and coronary heart disease, likely due to the combined effects of dehydration, blood viscosity, and thermoregulatory strain [52].

## Chemical Pollutants

Chemical pollutants represent a growing health risk with increasing evidence pointing to important effects on CVD and metabolic diseases. There are > 350,000 chemicals registered for use globally, according to the United Nations Environment Program (UNEP) and the Organization for Economic Co-operation and Development (OECD). Of these, < 10,000 have any characterization of potential health effects. Even fewer have undergone comprehensive toxicological evaluation that includes long-term and low-dose exposure assessment. Emerging research has identified several generally recognized as safe (GRAS) chemicals that can adversely impact the gut microbiome and contribute to metabolic dysfunction. Such chemicals include widely used emulsifiers/preservatives (e.g. carboxymethylcellulose and polysorbate 80), artificial sweeteners (e.g. sucralose, saccharin, and acesulfame potassium), and certain food colorants (e.g. titanium dioxide), all of which have been implicated in the alteration of the gut microbiome, impaired glucose tolerance, and the promotion of low-grade inflammation/immune modulation linked to obesity and insulin resistance [54, 55].

Perfluoroalkyl and polyfluoroalkyl substances (PFAS) constitute an important subgroup of chemical pollutants which have a particularly notable contribution to CVD risk given their pervasive use and longevity in the environment. Chemically, PFAS feature a highly durable carbon–fluorine bond and are easily water-soluble as a weak acid. Due to these properties, PFAS are detectable almost universally in US population [56]. Several high-quality studies have demonstrated associations between PFAS and hypertension in various populations with a broad range of exposure [57, 58]. Recent data has even linked PFAS exposure to the risk for acute coronary syndrome in certain populations [59].

Micro and nano-plastics and plastic-associated compounds represent an additional subgroup of chemical pollutants with important cardiovascular and metabolic impacts [60–63]. Notably, these pollutants have been closely linked to endocrine dysregulation and may be contributing to the national obesity epidemic [60]. Bisphenol A (BPA) is a well-recognized plastic-associated compound that, despite being regulated, continues to contribute to cardiometabolic disease risk given its durability and pollution of natural water sources [60, 64]. Several analyses using the United States National Health and Nutrition Examination Surveys (NHANES), an ongoing cross-sectional study, have linked BPA exposure to various CVD outcomes [61].

## Contaminant Metals

The deleterious health effects of exposure to toxic metals have long been known, with the ancient Roman author Vitruvius associating the use of lead water pipes to adverse health effects centuries ago. Despite this, lead and other toxic metals continue to be used in several household and industrial applications. A significant burden of the adverse health effects of toxic metal exposure is manifested as CVD. This was acknowledged by the American Heart Association in a 2023 Scientific Statement [65].

Despite the established negative health impacts of lead exposure, global production increased from 8 million tons in 2006 to 12 million tons in 2018 [65]. Current routes of exposure include lead-based paint, primary and secondary exposure to both conventional and e-cigarettes, lead-acid batteries, water pipes, and various household goods, including toys, cosmetics, and electronics. Importantly, the continuing health impact is seen with low level exposures [65]. One of the primary mechanisms by which lead, a divalent cation, can enter cellular pathways and contribute to disease is by serving as a substitute for other divalent cations, such as calcium, resulting in deleterious downstream effects. The Global Burden of Disease Study found that the number of global stroke deaths and disability-adjusted life years (DALY) attributable to lead exposure in 2019 were 305.27 and 6738.78 thousand, respectively, which constituted approximately 5% of the total number of stroke deaths and DALYs in 2019 [66].

Cadmium is another important contaminant metal that contributes considerably to residual CVD risk [2]. Similar to lead, it’s used in a number of household and industrial applications, including batteries, pigments, ceramics, and glassware [65]. Serving as a substitute for zinc in various cellular pathways, cadmium is known to cause endothelial injury, promote the release of inflammatory cytokines, and contribute to oxidative stress [65]. A linear dose response relationship between degree of exposure to cadmium and CVD risk is seen (at 1 μg/L, RR 2.58, 95% CI: 1.78–3.74) [67].

Arsenic is a toxic, carcinogenic metalloid most commonly encountered in contaminated drinking water, but also found in polluted food and air [2, 65]. Associations between arsenic exposure and clinical or subclinical CVD have been demonstrated in several cohorts, including a group of 245 participants aged 9 to 11 years where arsenic exposure was associated with both carotid intima media thickness and concentric left ventricular hypertrophy [68]. Importantly, arsenic has been shown to alter lipid homeostasis and promote the formation of foam cells, a principal component of atherosclerotic plaque [65]. In a study published by Domingo-Relloso et al., the role of epigenetic dysregulation by arsenic exposure was highlighted. Among 2321 participants of the Strong Heart Study, blood DNA methylation and urinary arsenic levels were measured. Differentially methylated positions (DMPs) that served as potential mediators for CVD incidence and mortality as well as arsenic-related health effects were identified. Of the 20 DMPs associated with CVD incidence and 13 DMPs associated with CVD mortality, 11 were found to be associated with incident CVD in three prospective cohorts (Framingham Heart Study, Women’s Health Initiative, and Multi-Ethnic Study of Atherosclerosis), many of which were also associated with arsenic exposure [69].

## Residual Environmental Risk

**Fig. 2:**
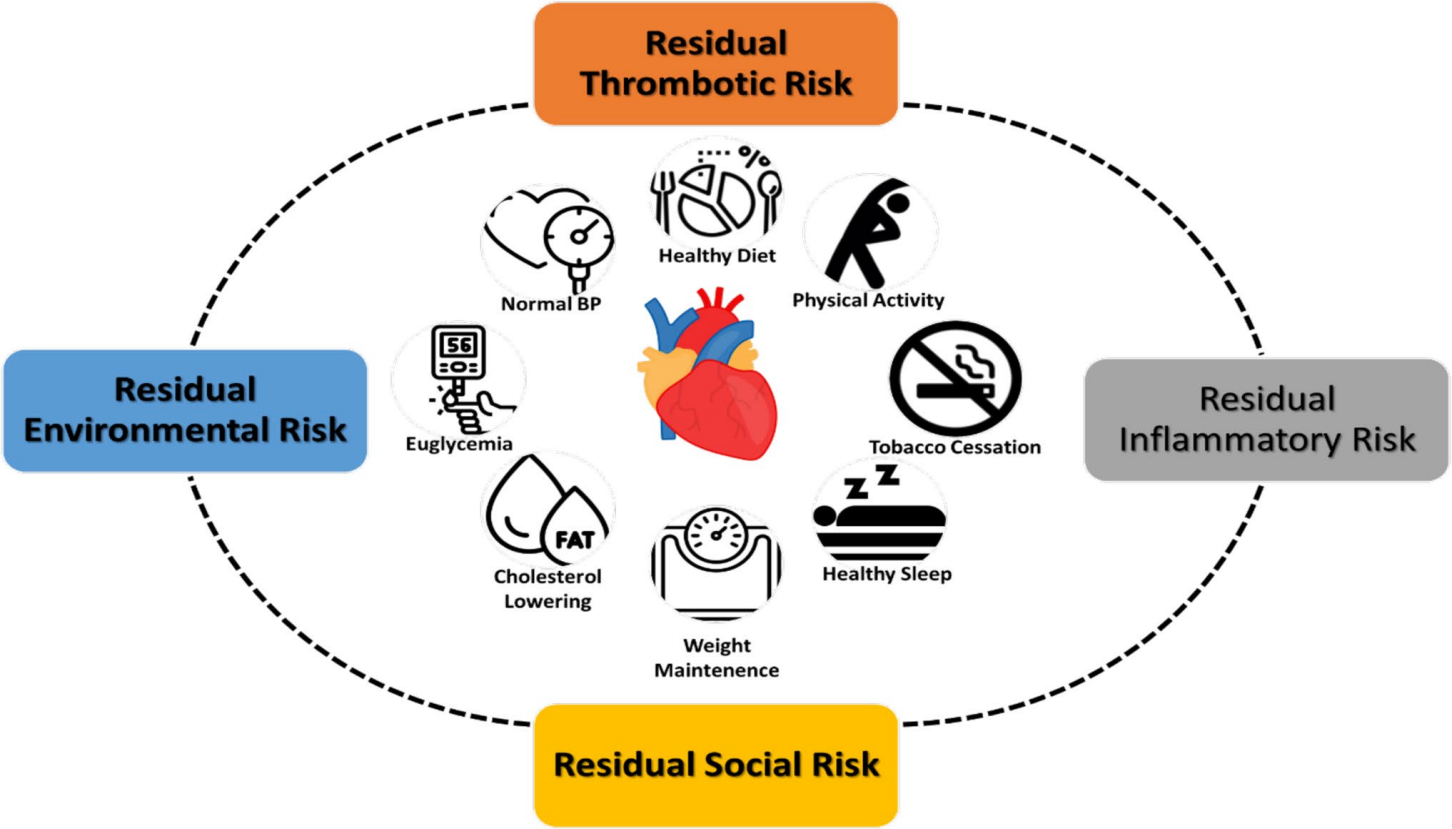
Residual risk paradigm in patients with cardiovascular disease. Traditional risk factors are shown centrally, while residual risks are shown surrounding these factors to highlight their important interaction with traditional risk factors. Residual risks include thrombotic and inflammatory risks which are well-known contributors to cardiovascular disease risk, as well as social and environmental risks which have received growing recognition as important determinants of cardiovascular outcomes. This figure was reproduced with permission from Rajagopalan S et al. Eur Heart J. 2023;44(44):4612-4614

The concept of residual cardiovascular risk refers to the contribution of all environmental factors to continuing cardiovascular risk after all standard modifiable risk factors (SMuRFs) have been addressed (Fig. 2) [70]. Addressing residual CVD risk may require a re-examination of cardiovascular risk as currently perceived by healthcare professionals. Many environmental factors, such as chemicals, metals, or pollution, are not on physician radars despite their acknowledgement that these factors often pose the same if not higher risk than many traditional risk factors to cardiovascular health. Furthermore, given that many are modifiable, residual cardiovascular risk factors present avenues to lower risk once they are identified.

For instance, each 20 mg/m^3^ increase in ambient particulate matter air pollution exposure is associated with 26% increase in major adverse cardiovascular events in patients with coronary artery disease undergoing percutaneous coronary intervention, independent of clinical factors.[5]The same elevation can also cause 1–2.0 mmHg increase in systolic blood pressure (SBP). At an individual level and in patients with low baseline absolute risk, this increased risk may be largely inconsequential, but for those who are susceptible or pre-disposed it may result in a substantial increase in cardiovascular events. Residual environmental risk can help explain substantial geospatial variance (> 50%) in CVD but also help address differences in personal risk for CVD.

## Moving from Individual Exposures to the Exposome

**Fig. 3:**
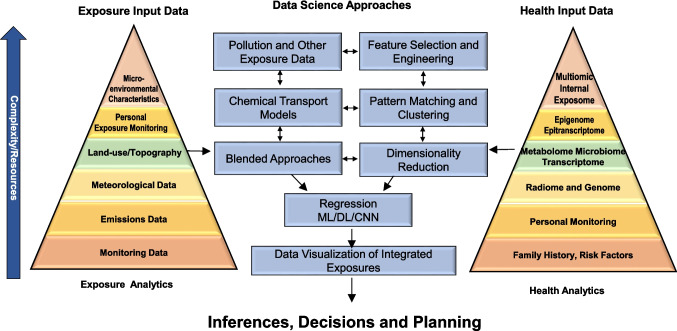
Data Science Approaches to Derive Exposomic Signatures. ML, machine learning; DL, Deep learning; CNN, convolutional neural networks. This figure was reproduced with permission from Khraishah H et al. Circ Res. 2024;134(9):1083-1097

Addressing the sum totality of exposures may warrant a departure from the current “one exposure at a time framework” to simultaneous examination of multiple exposures that impact humans throughout most locations in the modern world in a near continuous and additive (or even synergistic) fashion [13]. The integration of climate, environmental, social, and health data into common platforms and the use of machine learning and artificial intelligence (AI) to explore climate and human health effects provides an unprecedented opportunity for health impact assessment (HIA) and policy (Fig. 3).

In the field of exposure science, new sensor technologies offer the promise of portable and/or wearable monitors that can capture multiple human microenvironments in an integrated assessment for one or multiple chemicals. Such monitors can be combined with cell phone location information and video capability to gather extensive information about neighborhood level and/or personal environmental exposures. Place-based biomonitoring could be used to develop geospatial cumulative exposure profiles. Modern analytical methods, including liquid chromatography-mass spectrometry, can detect > 30,000 small molecules in human serum in one run [71]. However, these untargeted approaches may miss the vast majority of chemical exposures. Targeted approaches, including metabochips and immunochips, increasingly offer viable alternatives that are also cost-effective [71]. Epigenetic changes are typically measured using DNA methylation in which samples may represent methylome (EPIC or BeadChip) or the whole genome (whole genome bisulfite sequencing). "Enviromics" refers to high-throughput Omics techniques used in exposome studies applied to a broad variety of health conditions including cardiovascular disease [72, 73]. Exposome studies such as HELIX [74, 75], PACE [76, 77], UK Biobank [78], and birth cohorts represent important assets to dissect complex exposomic impacts especially during vulnerable phases, including during development and early childhood. Additional promising projects include EXPOSOMICS consortium [79, 80], HERCULES [81], HEALS [82], and the EUROPEAN EXPOSOME NETWORK [83].

## Important Precepts in Environmental Risk Assessment

The appropriate sequence of integration of any risk factor involves awareness amongst both patients and physicians of the existence of such a factor(s), identification or detection of at-risk individuals and/or communities, and finally the implementation of risk-mitigating strategies/interventions.

## Awareness and Detection

Awareness of environmental pollutants as a health threat for CVD continues to be poor amongst both patients and physicians [84, 85]. Most environmental risk factors are invisible and are often poorly understood in terms of their cardiovascular health risks [14]. Even awareness of the effects of visible, well-known, and easily interpretable risk factors, such as air pollution, poses significant challenges. The health sector is seen as a key player in disseminating evidence on health risks and teaching exposure reduction strategies; thus, the current lack of emphasis on environmental risk factors and the lack of engagement and emphasis on linking.

environmental exposures with health risks remains a major barrier to spreading awareness. Many toxic exposures are currently not specifically labeled as cardiotoxins, despite their strong connections to CVD, and are instead often characterized as carcinogenic. Tobacco smoke, as an example, is often labeled as a carcinogen but not labeled as atherogenic. Community awareness and the dissemination of concise, accurate, evidence-based information are fundamental to catalyzing multisectoral action and are currently nascent [49].

## Susceptibility and Vulnerability

Increased susceptibility defines individuals (e.g., elderly adults, those with diabetes, patients with prior cardiovascular disease, immunosuppressed individuals, etc.) who are at higher risk for adverse health effects than the general population when facing the same level of exposure [86]. Vulnerability from an epidemiologic and policy standpoint generally refers to a broader construct of populations who face disproportionately high exposures and hence a greater burden of health effects. The World Health Organization (WHO) specifies vulnerable populations based on innate and acquired environmental, social, and behavioral factors [87]. Most regulatory policies are designed to protect the most vulnerable among the general population [88]. From a population perspective, it is imperative to identify vulnerable populations by individual or neighborhood characteristics, as the risk posed by many environmental risk factors are pervasively personal, requiring detailed characterization of the exposures in one’s personal environment (e.g., chemical exposures). This has been a growing area of emphasis in the air pollution and cardiovascular literature, driven by the increased emphasis on environmental justice [86, 88–90].

## Risk Assessment Approaches

Most environmental factors are often not considered as part of risk assessment for CVD. Prior cardiovascular guidelines recognize the contribution of environmental factors broadly in the pathogenesis of CVD, but only factors pertaining to diet and lifestyle had been explicitly acknowledged until recently [91]. The 2024 European Society of HTN (ESH) Guidelines on HTN, for the first time, recognized the importance of environmental risk factors, such as air pollution, but stopped short of integrating explicit environmental assessments and considerations into HTN management [92]. The 2025 Hypertension Guidelines finally acknowledges air pollution and metals (Pb, Cd, and As) as significant contributors to hypertension [93]. While nationally available indices, such as the Air Toxics Registry, provide area level (census tract) risk assessment for a range of cancers, no such integrated tools exist for CVD. Most available tools such as the Environmental Justice Screening Method (EJSM) from the California Air Resources Board and California Communities Environmental Health Screening Tool (CalEnviroScreen) map area level measures of cumulative health impacts for a range of health, environmental, and social vulnerability measures [13, 94]. Despite fine geographic resolution for some pollutants such as PM_2.5_, given the limited scope of exposures currently measured, these measures may not provide personalized assessments but can be helpful in refining personalized risk assessment tools. The AHA-PREVENT equation, while championing a race agnostic construct for derivation of risk using social deprivation index (SDI), falls short in improving risk discrimination of at-risk populations [95]. Newer approaches that represent better estimates of pollutant exposures, including geospatial measures such as street views, satellite views, or even unmanned aerial vehicles can provide superior geospatially resolved estimates of pollutant exposures [96–99]. Additionally, the use of personal monitoring devices can augment many area level measures and improve their performance, but this remains an area of research.

## Clinical Approach for Risk Assessment

Given the growing recognition of environmental risk factors and their contribution to traditional risk factors, such as hypertension and obesity, it is timely to explicitly incorporate exposures like air pollution into cardiovascular risk assessment/management guidelines [7, 9]. A structured clinical approach to environmental risk assessment could meaningfully inform decision-making and targeted interventions. [100] At present, integrated exposure–response (IER) curves that provide quantitative risk estimates for cardiovascular events of selected pollutants—most notably ambient air pollution, temperature, noise, and lead— can serve as initial templates for progressively developing comprehensive exposure–response relationships across a broader spectrum of environmental factors.

In the interim, clinicians can use existing evidence to identify individuals at elevated risk of pollution-attributable cardiovascular disease and offer tailored recommendations, ranging from personal mitigation strategies (e.g., air filtration, behavioral modifications) to advocacy for community-level interventions. Geospatial risk maps, derived by translating available exposure–response relationships into geospatially encoded exposure maps, can help identify populations with elevated CV risk. Integration of environmental, climate, social, and health data within unified platforms, enhanced by machine learning and AI, provides an unprecedented opportunity for dynamic risk stratification, health impact assessment, and policy translation [101]. While stronger evidence from prospective clinical trials is awaited to refine risk mitigation strategies, an interim framework can be proposed. This staged approach, starting with pollutants for which exposure–response data are available and expanding upon them as evidence for other pollutants emerges, lays the groundwork for a comprehensive, multi-exposure environmental risk assessment paradigm in cardiovascular medicine.

**Fig. 4:**
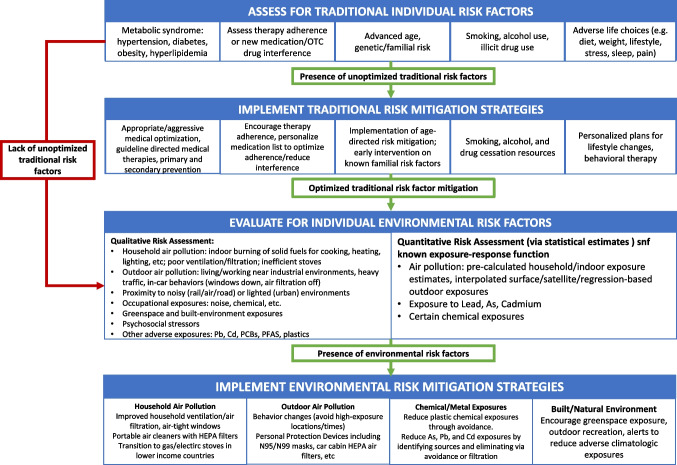
Suggested algorithm for incorporating environmental risk factors in clinical practice of cardiovascular disease prevention. Pb, lead; Cd, cadmium; PCB, polychlorinated biphenyls; PFAS, perfluoroalkyl substances. Adapted from Rajagopalan S et al. Hypertension. 2025;82(4):561-572. 10.1161/HYPERTENSIONAHA.124.18733 and Hadley M et al. 10.1161/CIRCULATIONAHA.117.030377

Key steps in the clinical assessment of environmental origins of CVD include evaluating patients for significant exposures to environmental stressors, including living in locations with high levels of ambient air pollution, heavy traffic-related particulate matter (PM), seasonal temperature changes, and chemical exposures at work or home. Patients reporting a lack of traditional risk factors should prompt clinicians to investigate potential environmental contributors Figures 4 Provides a broad approach

## Risk Intervention for Environmental Risk Factors

### Policy Interventions

Policy plays a central role in reducing cardiovascular harm from environmental exposures. The structural drivers of nearly all environmental exposures stem from energy, transportation, food, and urban systems that only policy can transform at scale**.** Coordinated efforts across all levels of government are needed to embed and enforce policy and de-risk environmental exposures. In the 1970 s, landmark U.S. policy actions, such as the Clean Air Act of 1972, the 1973–1975 phase-down of lead in gasoline under the Clean Air Act, and finally the 1978 federal ban on lead-based paint in residential housing, drove dramatic reductions in air pollution and lead exposures. These policy measures were guided by epidemiological and toxicological evidence that associated air pollution and lead with health effects. By targeting sources, setting limits, and pairing regulation with industry compliance timelines, ambient air pollution levels and lead levels in the U.S. population fell more dramatically over two decades to levels that hadn’t been experienced in the United States for over 100 years.

These examples showcase how coordinated science-based policy, regulatory limits, industry transition strategies, and sustained enforcement can yield rapid, large-scale reductions in environmental exposures, which ultimately finance themselves and provide a massive upside. The removal of lead from gasoline generated an estimated economic benefit in the past 40 years of over $8 trillion USD [102]. Every dollar invested in air pollution control in the USA since the passage of the Clean Air Act in 1970 has yielded a return of $30 USD (range, $4–88) [103]. Thus, the employment of cost-effectiveness analyses to guide investment in equitable, heart-healthy environments should be prioritized. Broad governmental policies should support HIA tools and prioritize interventions in under-resourced communities given that they bear the burden of environmental exposures. Policy solutions work best when combined to leverage synergistic effects—e.g., food policies paired with exercise promotion. Table 1 provides several pragmatic policy initiatives that, through reduction of one or more exposures involving provisioning systems (food, water, transport), improve cardiometabolic health [49]. Advancing education and advocacy initiatives for clinicians and the public and training a next generation of physician-advocates fluent in both cardiovascular health and environmental sustainability could have far-reaching consequences. By integrating health, sustainability, and equity into urban planning and policy, governments can shift the paradigm from risk recognition to transformative risk reduction**,** laying the groundwork for healthier, more resilient populations.

**Table 1:** Public Policy Solutions to Address Urban Environments for Cardiovascular Health at Different Levels of Government

Issue	Principle or pathway to support heart-health	Exposures Impacted	Examples of Specific Public Policy Solutions
Integrated Spatial Planning	Integrating land use planning and transportation planning departments Cardiovascular health as an explicit city planning goal can highlight its importance. Very few cities have explicit health-focused actions in national transport policy	Air pollution, noise, green space, and stress	Adoption of comprehensive (growth, master) plans; zoning and development ordinances and regulations; and (critically) permitting practices that prioritize: 1. Compact and mixed development patterns 2. Inclusion of diverse and affordable residential stock proximate to essential needs, services, employment, and green space 3. Reduction of sprawl and protection of open space and high value arable land, especially that able to provide quality food to nearby urban populations 4. Permanent protection of critical water resources
**Mixed Use**			
*Connectivity*	Requirements for street connectivity, pedestrian and cycling infrastructure, access to public open spaces (including parks)	Physical activity through increased active transportation (walk, bike, transit) & recreational walking & cycling	1. Ensuring access to public transportation within easy walkable distances 2. Active transportation options that are convenient 3. Mandatory laws promoting open access recreational areas to promote physical activity 4. Taxes on private transport and/or restricted access streets
*Zoning provisions*	Consider enabling language to encourage urban agriculture. While zoning codes are often not written to specifically prohibit urban agricultural activities, the absence of express permission deter potential growers	Access to healthy food sources through more equitable, mixed development patterns	1. Specify where and how city and public land can be used for personal or community production 2. Allow on site sale of healthy produce 3. Establishing urban garden districts 4. Incentives for urban farming and public green space
**Provisioning Systems**			
*Energy Sector*	Transitioning energy systems toward clean energy and low carbon goals to reduce air pollution	Air pollution, noise, green space, and stress	1. Public Investment in Renewable Energy at the local, regional level 2. Develop and Implement strategies to phase out Coal-Fired Power Plants by 2030 3. Support transitions to electric mobility and heating systems, linked to low carbon supply 4. Deep incentives for climate technologies that support mitigation and resilience 5. Update and Implement Current Federal Ambient Air Quality Standards from the current 12 µg/m3 annually to < 10 µg/m3
*Energy access in cities and decarbonization*	Integrated electrification strategies and planning program. Technical assistance and operational support to governments for geospatial electrification planning, pipeline development and implementation, preparation and minigrid investment portfolios Improving global electrification platform and applications Developing data standards for electrification planning in coordination with key partners	Air pollution	1. Facilitating clean energy access in cities through financial incentives 2. Improving energy grids and decentralizing energy infrastructure 3. Increasing electric car charging outlets
**Mobility**			
*Metropolitan planning*	Metropolitan planning for transit-oriented development stimulates active mobility and equitable access to green/public spaces and health care facilities Integrate Complete Streets policies in all roadway construction, repair, and maintenance Cardiovascular health as an explicit city planning goal can highlight its importance. Few cities have explicit health-focused actions in national transport policy	Physical activity through increased active transportation (walk, bike, transit) and recreational walking and cycling	Planning and transport agencies to award infrastructure funding according to the following: 1. Compact development communities and mixed land use areas 2. Full accommodation of active transport modes (walk, bike, and transit) and targeted reduction in motorized vehicle miles traveled 3. Exceptional service to vulnerable (and often historically neglected) populations based on age, race, ethnicity, income, sex, disability, car ownership, health status Adoption of prescriptive Complete Streets policies that: 1. Require context appropriate accommodation of all modes (walk, bike, transit, motor vehicle) in all roadway projects 2. Adoption of state-of-the-art multimodal design guidance (eg, the US National Association of City Transportation Officials design guides) 3. Application of Complete Streets design principles in routine painting and maintenance programs 4. Explicit and rigorous requirements limiting exceptions to fully multimodal designs
*Active transportation* *Demand management*	Participation targets for walking and cycling (eg, percentage mode share) Managing the demand for car travel influences the appeal of driving relative to other transport modes, with consequences for health	Reduced exercise, social stress Air pollution	1. Active Transport Alternatives. High Volume and Efficient Active Transportation Alternatives such as High-volume pedestrian walkways 2. Congestion Pricing: Introduce congestion pricing to discourage car use during peak hours. Use revenue generated to fund public transportation and active transport 3. Carpooling and Ridesharing Incentives: Incentives for carpooling and ridesharing, such as preferential parking or reduced tolls 4. Park-and-Ride Facilities: Develop park-and-ride facilities to encourage public transportation for final leg
*Policy decisions for connecting the city*	Options for formalizing existing transport systems and trade-offs	Increased physical activity through increased active transport modes Reduction of congestion, air and noise pollution; stress reduction	1. Comprehensive Transportation Demand Management (TDM) programs and policies that shift behavior toward routine walking, cycling, and transit use, and away from single occupancy vehicles. These include (but are not limited too): 2. Worksite and school incentives for the use of the active modes such as financial rewards; subsidized/free transit; schedule flexibility; elimination of free parking; accommodations for bicycle commuters; etc 3. Urban policies such as congestion pricing of roadways and parking; urban mobility aids (shared bikes & scooters); decoupling parking from development requirements and planning for complementary parking uses
*Distance to Transit/Destination*	Decreasing distance between transit and destination (15 min or 30 min) and enabling active modes to access transit choices		1. Enact guidelines for easy access cities where people can conveniently access jobs and services within 20–30 min by public or active transport, 7 days a week 2. Implement integrated ticketing systems that allow seamless transfers between different modes of transportation, including buses, trains, and bicycles
**Housing and Access**	Support equitable development and preservation of affordable housing	Increased physical activity through active transportation; enabled by equitable housing availability proximate to daily needs and employment centers Stress reduction Access to healthy food sources	1. Strengthen prohibitions on discriminatory home loan lending practices to ensure that people of all races, ethnicities, and backgrounds have access to home ownership 2. Increase planning, zoning, and regulatory support for alternatives to single family homes and high-density apartments. Near to daily needs, services, healthy food, and employment provide the so-called missing middle, such as row houses & town homes, multi-plexes, cottage clusters and tiny homes with shared green space, accessory dwellings, shared housing, and myriad evolving options
**Food Systems**	Food systems should transition to ensuring nutrition security through equitable and stable availability, access, affordability, and use of foods and beverages that promote health and well-being		1. Implementing Robust Food Service Guidelines and Procurement Standards in Government Buildings, Institutional Feeding Programs, and Private Sector Food Service 2. Support sustainable, climate-smart urban-regional agriculture and associated farmers markets and food carts to increase access to nutritious foods
**Green infrastructure**	Spatial distribution of parks can enable access to nature, leisure and active mobility, and increased tree canopy coverage to reduce heat stress	Temperature, Noise and Air Pollution, Stress	1. Education campaigns 2. Aggressive reforestation and green infrastructure in urban areas to reduce heat island effects 3. Spatial distribution of parks can enable access to nature, leisure and active mobility, and increased tree canopy coverage to reduce heat stress 4. Public private partnerships to increase urban gardens in neighborhoods 5. Biophilic buildings with green walls and roofs and small pocket parks
**Water Supply**			
*Wastewater Management*	Improve water quality by reducing pollution, eliminating release of chemicals and plastics to water bodies Reduce exposure to toxic metals including lead in urban hot spots, often in underserved communities	Water Pollution	1. Automated Networks for water quality and surveillance of release of chemicals and plastics 2. Moratorium on use of plastics and plastic associated chemicals 3. Public Education 4. Mandate adherence to safety thresholds in City water supplies with specific attention to Lead, Cadmium, Nickel and Arsenic 5. Recycling of wastewater and stormwater
*Construction materials, chemicals, plastics, and waste management*	Use of low carbon or “green” infrastructure including cement, building materials Promotion of circular design of products and materials for reuse, remanufactured or recycled with the intent of retaining in the economy for as long as possible along with the resources they are made of, as well as minimizing the generation of waste		1. Incentives to promote green construction and materials 2. Ban and eliminate single use plastics in urban environments 3. Pay to use for plastics that are recyclable 4. Specific mandates on disposal of plastics in urban environments such as restaurants, groceries, and public arcades
**Specific Exposure Mitigation**			
*Air Quality*	Reduce exposure to ambient air pollution for all people living in the United States and globally	Air Pollution	1. Emission Control. Implement and enforce stringent emission standards for industries, vehicles, and power plants. Regularly update these standards to reflect advancements in technology and understanding of air pollution 2. Public Transportation Improvement. Invest in and expand public transportation infrastructure to reduce the reliance on individual vehicles. Promote the use of electric and hybrid vehicles through incentives and subsidies 3. Vehicle Emission Controls. Implement and enforce vehicle emission testing programs. Introduce vehicle emission standards and promote the use of electric and hybrid vehicles 4. Industrial Emissions. Enforce strict regulations on industrial emissions and provide incentives for companies to adopt cleaner production technologies. Implement monitoring systems to track and control emissions from industrial sources
*Noise Pollution*	Reduce noise exposures in urban environments	Noise Pollution	1. Enact noise guidelines in urban environments that are consistent with global standards (e.g. Road noise < 53 dB L_den_ and < 45 dB L_nignt_) 2. Harmonize solutions that reduce noise with other key provisioning systems such as buildings, green infrastructure and transportation solutions that may aid in reducing ambient noise
*Adverse Temperatures*	In populations exposed to high ambient temperatures, strategies to protect populations from excess indoor heat should be developed and implemented	Temperature	1. Education campaigns 2. Aggressive reforestation and green infrastructure in urban areas to reduce heat island effects

Reproduced from: Rajagopalan S, Ramaswami A, Bhatnagar A, Brook RD, Fenton M, Gardner C, et al. Toward Heart-Healthy and Sustainable Cities: A Policy Statement From the American Heart Association. Circulation. 2024;149(15):e1067-e89

In many countries where political will and policy shifts could be fickle, action is frequently linked to incentives in the system. An example of how lack of appropriate incentives can delay policy shifts was exemplified in a recent paper on crop burning, a major source of air pollution in South Asia, where burning of crops is frequent and contributes to as much as 40% of air pollution and a dense haze in cities in Northern India and Pakistan [104]. Although crop burning is illegal in India, a recent study showed that perverse crop burning responds to bureaucrat incentives: fires increase by 15% when wind is most likely to direct pollution to neighboring jurisdictions and decrease by 14.5% when it pollutes the jurisdictions that the bureaucrat lives in. Further, this study also showed that one log increase in utero exposure to pollution from crop burning raises child mortality by 30–36 deaths per 1,000 births, underscoring the importance of solving this public health problem with incentives for policy decisions [104].

### Personal Interventions

Many environmental exposures that are harmful to human health, merit recommendations that are common-sense, low-risk, easy-to-follow behavioral avoidance actions based upon logical considerations. The avoidance action involving a change in behavior must be extremely low risk for the ensuing benefit, (i.e., reduced exposures) and to outweigh any potential adverse consequences of the behavior itself. Examples of personal risk avoidance include avoidance of drinking water with high lead or arsenic concentrations by substitution for an alternate water source, or avoidance of traffic pollution by following an alternate route, lower in air pollution. In each of these instances, the alternative should be virtually cost and risk free. In many cases, the alternative may be risk free but not cost free. The cost of bottled water or filtration devices for metals, for instance may pose economic hardships, especially in communities most at risk.

#### Personal Measures for Toxic Metals

Given the preponderance of evidence supporting the role of lead, cadmium, and arsenic in CV disease, this section is tailored to these metals. Cross cutting common approaches include routine testing in high-risk groups (e.g., well water, old housing, occupational exposure). Personal exposure reduction strategies include avoidance of well water if high levels are detected, avoidance of smoking (active or passive: Cd), soil exposure reduction by removing shoes indoors, washing and soaking produce and grains thoroughly, and finally using filtration devices, such as reverse osmosis systems. These strategies can be highly effective at removing most dissolved metals.

#### Plastic Associated Chemicals and Microplastic/Nanoplastic Exposure Reduction

Currently, there are no universally adopted clinical or public health guidelines for reducing human exposure to plastic-associated chemicals (such as phthalates, bisphenols, and flame retardants) or to microplastics and nanoplastics (MNPs) [105]. Nevertheless, a growing body of evidence links these exposures to metabolic, endocrine, reproductive, and cardiovascular effects [61–63, 105]. At the individual level, avoidance strategies include, minimizing consumption of foods stored or reheated in plastic containers, choosing fresh or minimally processed foods over ultra-processed and packaged foods, and using glass, stainless steel, or ceramic alternatives. Favoring tap water with certified filtration systems (e.g., reverse osmosis, activated carbon) over bottled water, although controversial, may avoid perpetuating plastic consumption. Avoiding consumer products such as cosmetics, toothpastes, and personal care products that contain microbeads and reducing use of single-use plastics wherever possible are overall reasonable strategies that involve minimal cost and potential health and economic benefit.

#### Personalized Measures for Air Pollution

Although no personalized intervention to date has demonstrated a reduction in cardiovascular events, the causal association of PM_2.5_ would suggest that mitigation strategies that reduce particle concentrations should indeed reduce risk. There is good evidence that cardiovascular surrogate markers, such as blood pressure, heart rate variability, and endothelial function improve with lowering of PM_2.5_. An AHA document has extensively reviewed the evidence of lowering PM_2.5_ with benefits on multiple surrogates including blood pressure [106].

#### Portable and Central Filtration Interventions

Portable air cleaners (PACs) equipped with HEPA filters are widely available and have been shown to reduce indoor PM_2.5_ levels by 50–60% with effectiveness depending on use at appropriate flow rates, airtightness of the home, and other factors. A meta‐analysis of randomized double‐blind air cleaner trials showed that a 10 μg/m^3^ decrease in PM_2.5_ translated into a ~ 2‐mm Hg reduction in SBP, which is a more than threefold greater response compared to the known dose response relationship between PM2.5 and SBP [7]. Central HVAC systems with high-efficiency filters can also reduce indoor particles, but are highly dependent on building design, filter maintenance, and operational factors, with cardiovascular benefits remaining limited.

#### Personal Air Filtration Devices

Multiple studies using air-purifying respirators, particularly N95 or N99 masks, and have shown short-term improvements in blood pressure [7, 107]. These typically reduce particle inhalation by more than 95% when properly fitted. However, effectiveness wanes with poor fit, adherence, and when high levels of ultrafine particles and/or gases are prevalent. Less protective surgical or cloth masks offer partial filtration but lack evidence for cardiovascular benefit and may pose behavioral challenges, such as fostering a false sense of security [106].

#### Behavioral and Pharmacologic Strategies

Simple lifestyle strategies can help limit exposure to pollutants, provided knowledge regarding sources, routes of exposures, and prevalence of such exposures, which varies by pollutant type and setting. Avoidance of traffic-related pollution—such as rerouting commutes, exercising away from major roads, and using GPS-enabled “safe corridors” with lower air pollution exposure—may reduce exposure to ultrafine particles and gaseous co-pollutants. However, when no other options are available, the benefits of exercising in polluted environments may outweigh risks in healthy individuals, except in heavily polluted environments. Staying indoors with windows and doors closed with air conditioning may reduce exposure provided the air conditioning units filtration devices are clean. At present, no pharmacologic agent can be recommended specifically for pollution mitigation under any circumstance; however, maintaining evidence-based therapies for primary and secondary prevention of cardiovascular disease remains essential while research continues to identify targeted interventions.

#### Other Environmental Exposures

Reduction in noise pollution may be considered especially in patients living close to airports or highways that are continually exposed to high levels of noise.

## Conclusions and Future Directions

There is strong and consistent evidence that environmental risk factors are major drivers of CVD and cardiometabolic risk, based on emerging mechanistic and epidemiologic findings. With an expanding number of exposures, including a vast repository of chemical and new anthropogenic and climate change induced exposures, there is an urgent need for new knowledge on their cardiovascular and metabolic risk profile and for the incorporation of known pollutants into comprehensive exposomic measures of risk. The successful implementation of a true exposomic framework remains a challenge and will need satisfactory resolution of several issues, including data homogenization, standardization, and ultimately interpretability. The use of machine learning and AI may facilitate such an integrative approach and provides an unprecedented opportunity for HIA and policy. There is a critical need to incorporate environmental metrics and assessments in clinical trials as they provide an unprecedented opportunity to piggy-back these measures alongside conventional clinical metrics. Improvements in risk communication of common environmental risk factors, such as air pollution and air quality alerts that are health-based rather than pollutant level-based alone, are needed. Finally, successful integration of environmental risk exposures and exposomic frameworks into risk assessment are needed.

## Key references

- Rajagopalan S, Brook RD, Salerno P, Bourges-Sevenier B, Landrigan P, Nieuwenhuijsen MJ, et al. Air pollution exposure and cardiometabolic risk. Lancet Diabetes Endocrinol. 2024;12(3):196–208.
  - This review provides a thorough overview of the epidemiologic and mechanistic data linking air pollution with cardiometabolic risk.
- Khraishah H, Chen Z, Rajagopalan S. Understanding the Cardiovascular and Metabolic Health Effects of Air Pollution in the Context of Cumulative Exposomic Impacts. Circ Res. 2024;134(9):1083–97.
  - This manuscript discusses the impact of air pollution through the lens of exposomics and integrated risk assessment.
- Chen Z, Dazard JE, Deo S, Al-Kindi S, Rajagopalan S. Emerging AI tools for geospatially resolved cardiovascular risk. Nat Rev Cardiol. 2025.
  - This paper highlights the use of artificial intelligence tools to understand associations between geospatial factors and cardiovascular risk.

## Data Availability

Given this is a review article, all data cited is available as referenced.
